# Axial torsion of Meckel’s diverticulum causing acute peritonitis in the first trimester of pregnancy: a case report

**DOI:** 10.1186/s40792-019-0754-y

**Published:** 2019-12-05

**Authors:** Hiromitsu Nagata, Hiroyasu Nishizawa, Susumu Mashima, Yasuyuki Shimahara

**Affiliations:** Department of Surgery, Yamatokoriyama Hospital, 1-62 Asahichou, Yamatokoriyama, Nara, 639-1013 Japan

**Keywords:** Meckel’s diverticulum, Axial torsion, Pregnancy, Acute peritonitis

## Abstract

**Background:**

Meckel’s diverticulum is considered the most prevalent congenital anomaly of the gastrointestinal tract. Approximately 4% of patients are symptomatic with complications such as bleeding, intestinal obstruction, and inflammation, while axial torsion of Meckel’s diverticulum is rare, particularly in pregnancy.

**Case presentation:**

A 31-year-old woman in week 15 of pregnancy complained of epigastric pain, nausea and vomiting. Clinical diagnosis was severe hyperemesis gravidarum. Because the symptoms persisted during hospitalization, CT was performed and revealed dilated small bowel loops with multiple air-fluid levels. In the right mid-abdomen, there was a large part of air containing a cavity connected to the small intestine, which was considered a dilated bowel loop. Emergency laparotomy was performed and axial torsion of a large Meckel’s diverticulum measuring 11 cm was found at a few centimeters proximal to the ileocecal valve. Ileocecal resection including Meckel’s diverticulum was performed. The postoperative course was uneventful. At 40 weeks gestation, she had vaginal delivery of normal baby.

**Conclusion:**

The physiological and anatomical changes in pregnancy can make a straightforward clinical diagnosis difficult. Prompt diagnosis and management were needed in order to avoid significant maternal and fetal risks. The use of imaging examinations, especially CT examination, with proper timing may be helpful to prevent delay in diagnosis and surgical intervention. Here, we report the case of a patient with axial torsion of Meckel’s diverticulum in pregnancy. To our knowledge, axial torsion of Meckel’s diverticulum in the first trimester of pregnancy has not been reported in the English medical literature.

## Background

Meckel’s diverticulum (MD) is considered the most prevalent congenital anomaly of the gastrointestinal tract, affecting 2% of the population [1, 2]. MD is a true diverticulum of the small bowel, typically found within 100 cm proximal to the ileocecal valve. It results from incomplete obliteration of the omphalomesenteric duct [3]. Only 4% of patients with an MD develop complications such as bleeding, perforation, inflammation, and obstruction [4]. Axial torsion of MD is a rare complication [5–7]. Torsion of MD is the result of axial twisting around its base. The exact mechanism for this is unclear. The degree of torsion varies and can compromise diverticular circulation, leading to necrosis and perforation [8]. We hereby report a very rare case of axial torsion of MD in pregnancy.

## Case presentation

A 31-year-old woman in week 15 of pregnancy with pre-existing hyperemesis gravidarum complained of epigastric pain, nausea and vomiting. The patient had undergone a laparoscopic rt. ovarian cystectomy at 30 years old. The patient was hemodynamically stable with a temperature of 36.5 °C, pulse rate of 85/min, respiratory rate of 16/min, blood pressure of 101/52 mmHg and oxygen saturation of 100% on room air. The patient’s consciousness level was normal. The palpation of the abdomen revealed moderately hard and tender with rebound tenderness at the right upper quadrant. Laboratory examination demonstrated leukocytosis at 12,000/μl, 87% neutrophils, and normal hemoglobin, electrolytes, creatinine, AST, amylase, and C-reactive protein. The patient was first diagnosed with severe hyperemesis gravidarum and was admitted.

**Fig. 1:**
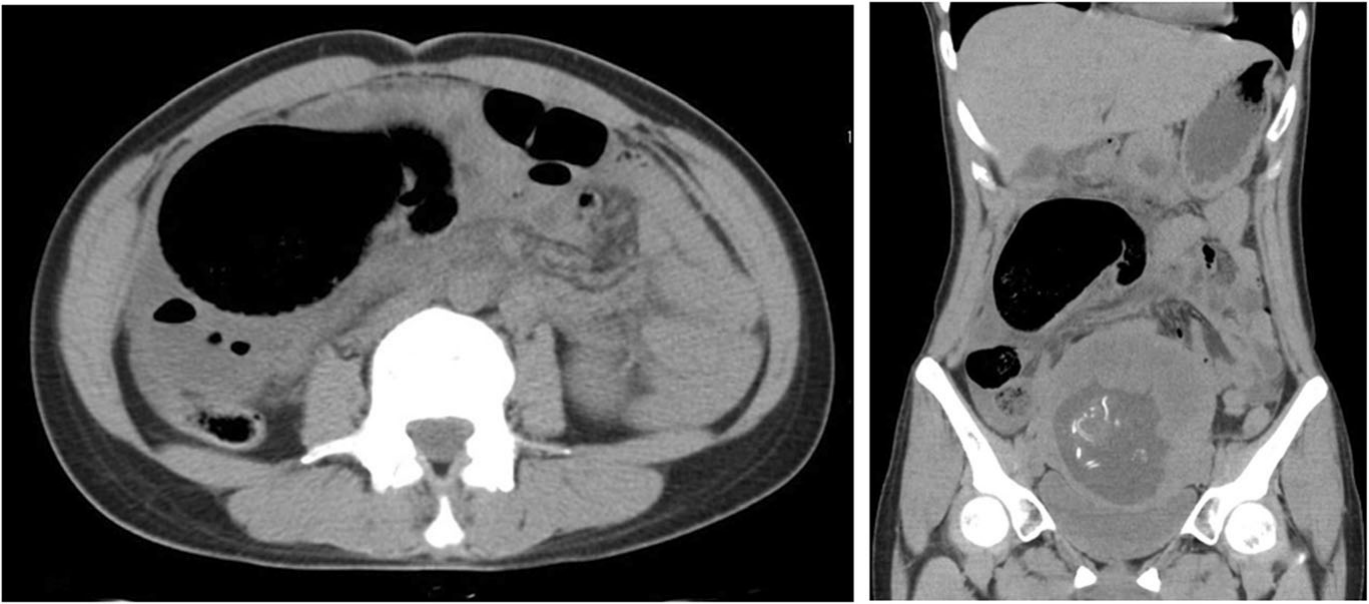
CT imaging of the abdomen (left, axial view; right, coronal view) In the right mid-abdomen, there was a large air containing cavity connected to the small intestine

**Fig. 2:**
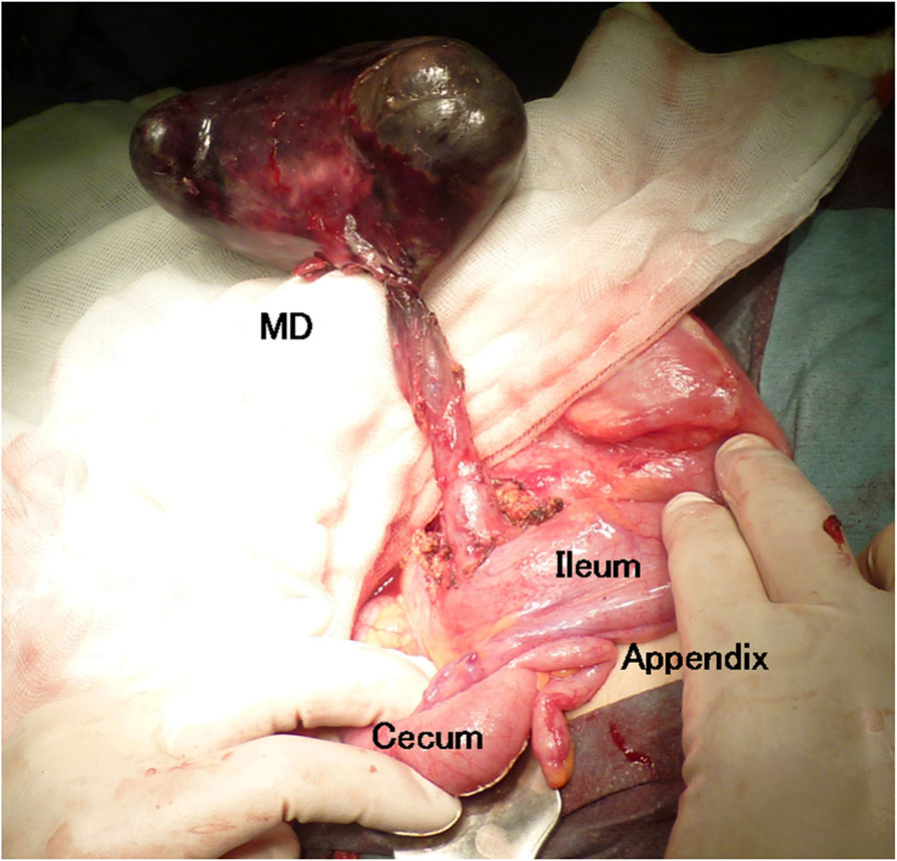
Intraoperative findings of the axial torsion of Meckel's diverticulum (MD)

**Fig. 3:**
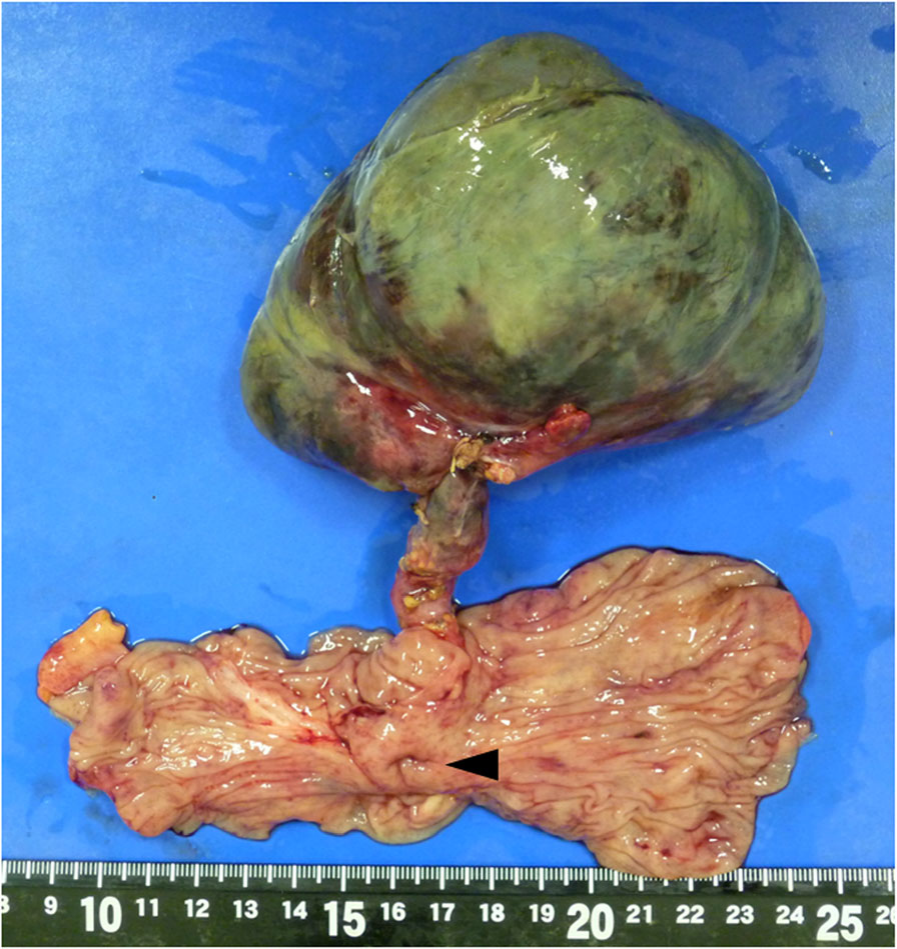
Gross findings of the resected bowel containing axial torsion of Meckel’s diverticulum (MD). The arrowhead reveals the orifice of Meckel’s diverticulum (MD)

Thereafter, the patient was commenced on trial of conservative treatment, including intravenous rehydration, antiemetics, and antibiotics. However, the patient presented with persistent abdominal pain. Thirty hours after the admission, laboratory examination demonstrated leukocytosis at 18,000/μl, 93.5% neutrophils, C-reactive protein of 13.7 mg/dl, fibrin/fibrinogen degradation products at 12.2 μg/ml (normal range, 0.0–5.0 μg/ml), D-dimer levels of 4.7 μg/ml (normal range, 0.0–1.0 μg/ml), base excesses of − 4.5 mmol/l (normal range, − 2.5–2.5 mmol/l), and nearly normal hemoglobin, electrolytes, creatinine, AST, and amylase. CT imaging of the abdomen revealed dilated small bowel loops with multiple air-fluid levels; in the right mid-abdomen, there was a large part of air containing cavity connected to the small intestine, which was considered a dilated bowel loop (Fig. 1). As acute peritonitis and strangulated ileus were diagnosed, an emergency laparotomy was planned. The peritoneal cavity was filled with a seropurulent fluid. Further exploration revealed a black and gangrenous MD, with axial torsion, measuring 11 × 8 × 5 cm, located 2 cm proximally to the ileocecal valve (Fig. 2). The ileocecal resection including MD was performed (Fig. 3). Postoperative histopathologic examination confirmed gangrene of MD without neoplasms. The patient recovered uneventfully and was discharged on the 16th postoperative day. At 40 weeks gestation, she had vaginal delivery of a normal baby.

## Discussion

This case report presents an extremely rare case of giant MD with torsion in pregnancy. A search of the English literature revealed only one case in the third trimester of pregnancy [9]. The etiology of the axial torsion of MD remains unclear. On the basis of the several articles, there are several risk factors: primary neoplasms arising within the MD [10], fibrous vitelline bands connected the MD to the abdominal wall [11], and the MD length and width of the base [8, 12]. The anatomical configuration, especially the diverticular length and base diameter are important predisposing factors. In particular, an elongated variant with a narrowed neck is far more likely to result in torsion [12]. In the case of the patient, the cause of axial torsion of the MD might be the giant head and narrow base as well as the bowel dislocation due to the gravid uterus.

Symptomatic MD is often difficult to diagnose because it mimics acute appendicitis, peptic ulcer, pelvic inflammatory disease, or gastroenteritis. In pregnancy, the diagnosis is even more difficult. The normal physiological and anatomical changes in pregnancy complicate the evaluation of acute abdomen. Normal symptoms of pregnancy like nausea, vomiting, abdominal, and pelvic discomfort overlap with the symptomatology of non-obstetric causes of acute abdomen. Many examination findings are altered as the gravid uterus displaces the other intra-abdominal organs, and signs of peritonitis may be absent due to lifting and stretching of the anterior abdominal wall. Leucocytosis, anemia due to hemodilution and tachycardia are normal findings in pregnancy. Therefore, imaging investigations for acute abdomen in pregnancy are important. Ultrasonography is the first-line imaging investigation for acute abdomen in pregnancy, though it has limited diagnostic capabilities in a pregnant patient with acute abdomen. The most useful diagnostic modality is CT examination [4], as can also be seen in the present report. MD is difficult to distinguish from the normal small bowel in uncomplicated cases on CT examination. However, a blind-ending fluid or gas-filled structure in continuity with the small bowel may be seen [13]. In this case, CT findings supported the emergency laparotomy even though radiation exposure by a CT scan is a major concern and the risks and benefits should be evaluated. The absolute risk of fetal effects is small at conceptus doses of 100 mGy and negligible at doses of less than 50 mGy [14]. CT examination of abdomen and pelvis rarely exceed 25 mGy. CT examination may be appropriate depending on the clinical situation, because the dose from a single-acquisition CT examination of the abdomen and pelvis poses a small risk to fetal health [14].

Surgical resection of symptomatic MD is the standard of care. Surgical options include simple diverticulectomy or ileal resection with the later procedure preferred when there is evidence of severe inflammation, perforation, or tumor [15]. In this case, the ileocecal resection including MD was performed in continuity with the adjacent inflamed intestinum by reason that tumors within the giant MD could not be completely ruled out.

## Conclusion

In view of the non-specific presentation of Meckel’s pathology in pregnancy, the management requires a multidisciplinary team approach consisting of a surgeon, an obstetrician and a radiologist in order to achieve a favorable outcome for mother and neonate.

## Data Availability

None.

## Abbreviations

AST: Aspartate aminotransferase
CT: Computed tomography
MD: Meckel’s diverticulum
